# Spontaneous intercostal artery bleeding occurring simultaneously in numerous vessels during antithrombotic therapy with mechanical circulatory support: a case report

**DOI:** 10.1186/s13256-024-04602-3

**Published:** 2024-06-16

**Authors:** Kazuto Ohtaka, Setsuyuki Ohtake, Yu Ishii, Saya Kaku, Yuta Takeuchi, Tomoko Mizota, Yoshiyuki Yamamura, Masaomi Ichinokawa, Tatsuya Yoshioka, Eiji Tamoto, Katsuhiko Murakawa, Koichi Ono, Tatsuya Kato

**Affiliations:** 1 Department of Surgery, Obihiro Kosei General Hospital, West 14 South 10, Obihiro, Hokkaido 080-0024 Japan; 2 Department of Thoracic Surgery, Hokkaido University Hospital, West 5 North 14, Kita-Ku, Sapporo, Hokkaido 060-8648 Japan

**Keywords:** Spontaneous intercostal artery bleeding, Transcatheter arterial embolization, Extravasation, Extrapleural hematoma, Case report

## Abstract

**Background:**

Intercostal artery bleeding often occurs in a single vessel; in rare cases, it can occur in numerous vessels, making it more difficult to manage.

**Case presentation:**

A 63-year-old Japanese man was admitted to the emergency department owing to sudden chest and back pain, dizziness, and nausea. Emergency coronary angiography revealed myocardial infarction secondary to right coronary artery occlusion. After intra-aortic balloon pumping, percutaneous coronary intervention was performed in the right coronary artery. At 12 hours following percutaneous coronary intervention, the patient developed new-onset left anterior chest pain and hypotension. Contrast-enhanced computed tomography revealed 15 sites of contrast extravasation within a massive left extrapleural hematoma. Emergency angiography revealed contrast leakage in the left 6th to 11th intercostal arteries; hence, transcatheter arterial embolization was performed. At 2 days after transcatheter arterial embolization, his blood pressure subsequently decreased, and contrast-enhanced computed tomography revealed the re-enlargement of extrapleural hematoma with multiple sites of contrast extravasation. Emergency surgery was performed owing to persistent bleeding. No active arterial hemorrhage was observed intraoperatively. Bleeding was observed in various areas of the chest wall, and an oxidized cellulose membrane was applied following ablation and hemostasis. The postoperative course was uneventful.

**Conclusion:**

We report a case of spontaneous intercostal artery bleeding occurring simultaneously in numerous vessels during antithrombotic therapy with mechanical circulatory support that was difficult to manage. As bleeding from numerous vessels may occur during antithrombotic therapy, even without trauma, appropriate treatments, such as transcatheter arterial embolization and surgery, should be selected in patients with such cases.

## Background

Intercostal artery bleeding arises from vessel fragility induced by various underlying conditions, including neurofibromatosis type 1, coarctation of the aorta, systemic lupus erythematosus, alcoholic cirrhosis, and trauma leading to aneurysm formation and rupture [1–6]. Intercostal artery bleeding can cause massive hemothorax, chest wall hematomas, abdominal wall hematomas, and paravertebral hematomas, some of which can be fatal [7–9]. It is often diagnosed using contrast-enhanced computed tomography (CT), which shows contrast extravasation [1]. While single-vessel bleeding is common, simultaneous bleeding from numerous vessels is rare [1, 10]. We report a case of a patient experiencing spontaneous intercostal artery bleeding from numerous vessels during antithrombotic therapy, making it difficult to manage.

### Case presentation

A 63-year-old Japanese man with type 2 diabetes mellitus who self-discontinued treatment and had a 20-pack-year smoking history was admitted to the emergency room owing to sudden chest and back pain, dizziness, and nausea. Although initial chest radiography and CT scans showed no anomalies, an electrocardiogram showed ST-segment elevation in leads II, III, and aV_F_. Subsequent coronary angiography (CAG) exposed three-vessel coronary artery disease, specifically myocardial infarction owing to right coronary artery occlusion. To address hemodynamic instability, such as systolic blood pressure failing to 50 mmHg, intra-aortic balloon pumping (IABP) and a temporary pacemaker were employed in addition to vasopressor administration. Percutaneous coronary intervention (PCI) was performed only on the right coronary artery, which was considered to be the culprit lesion. The patient was then transferred to the intensive care unit (ICU). Dual antiplatelet therapy (DAPT) with 100 mg of aspirin and 3.75 mg of prasugrel hydrochloride per day was initiated, and heparin was administered at a rate of 15,000 units per day for IABP, with checking the coagulation function every 8 hours.

**Fig. 1:**
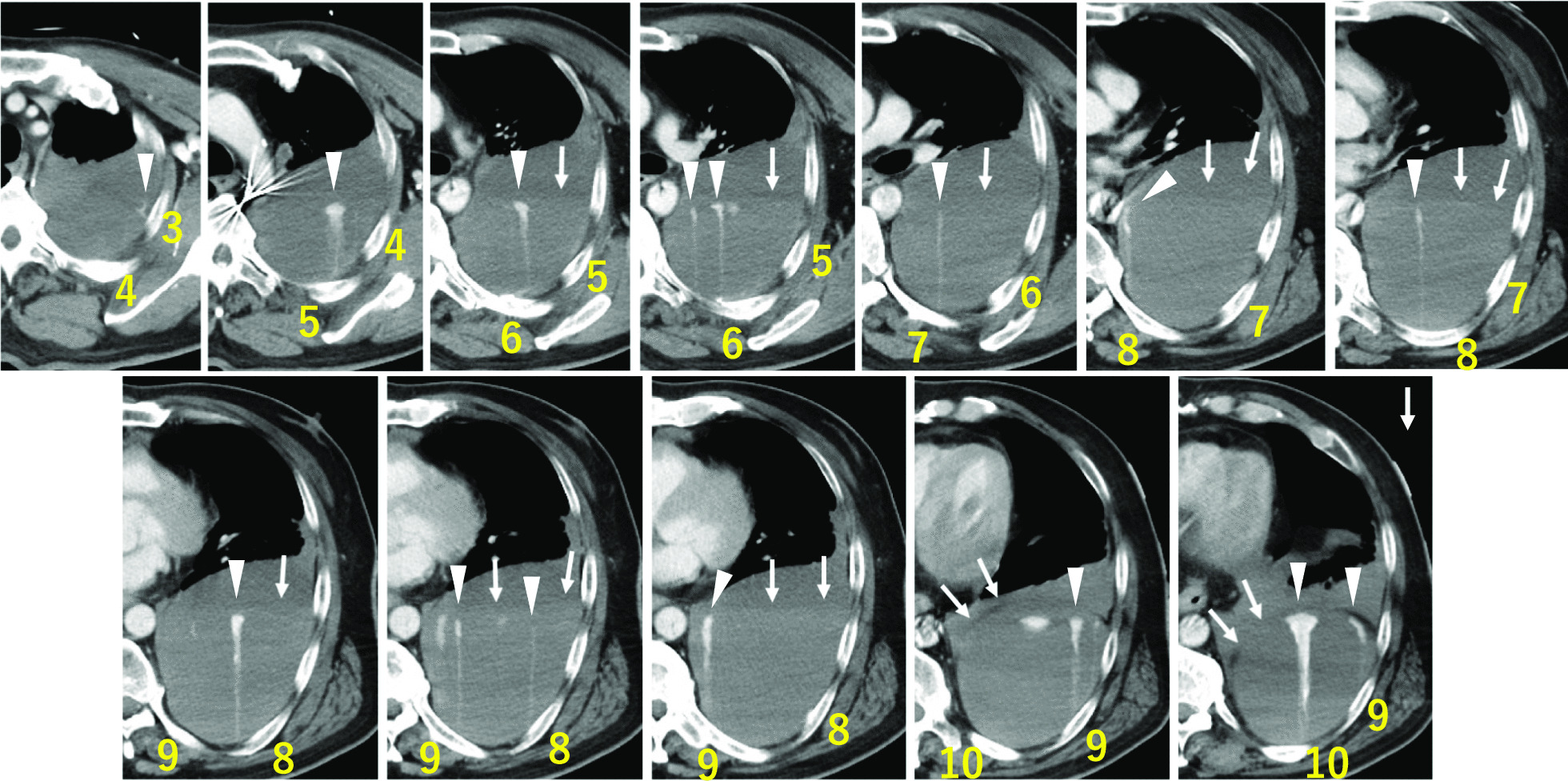
Contrast-enhanced computed tomography scan showing a large left extrapleural hematoma and multiple-vessel bleeding within the hematoma. Yellow numbers indicate the rib numbers. White arrows indicate the parietal pleura. White arrowheads indicate contrast extravasation

**Fig. 2:**
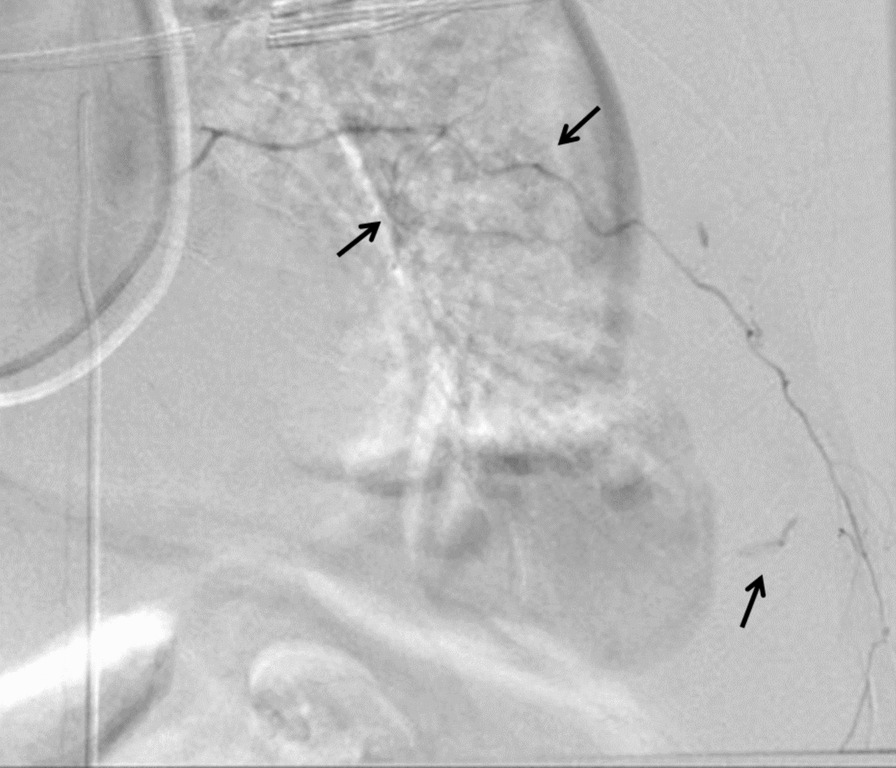
Emergency angiography showing contrast leakage from the left 6th to 11th intercostal arteries. Transcatheter arterial embolization was performed on these vessels. Black arrows indicate contrast extravasation

At 12 hours following PCI, the patient encountered left anterior chest pain, leading to reduced systolic blood pressure to 60 mmHg and hemoglobin levels to 70 g/L. Intubation was performed for pain relief after fluid and blood transfusions raised his blood pressure. The patient had a prothrombin activity rate of 89% and an activated partial thromboplastin time of 90.5 seconds. Contrast-enhanced CT scans after hemodynamic stabilization revealed a sizable extrapleural hematoma with 15 sites of contrast extravasation, that were suspected to be numerous intercostal bleeding (Fig. 1). While transcatheter arterial embolization (TAE) was planned, it was initially challenging owing to the position of the IABP balloon and the target intercostal artery. After IABP removal, veno-arterial extracorporeal membrane oxygenation (VA-ECMO) was introduced. A contrast leakage was noted in the left 6th to 11th intercostal arteries, leading to successful embolization (Fig. 2). Following TAE, an IABP was reinserted, and heparin was administered, maintaining clotting time. Hemodynamic stability was restored after blood transfusion.

**Fig. 3:**
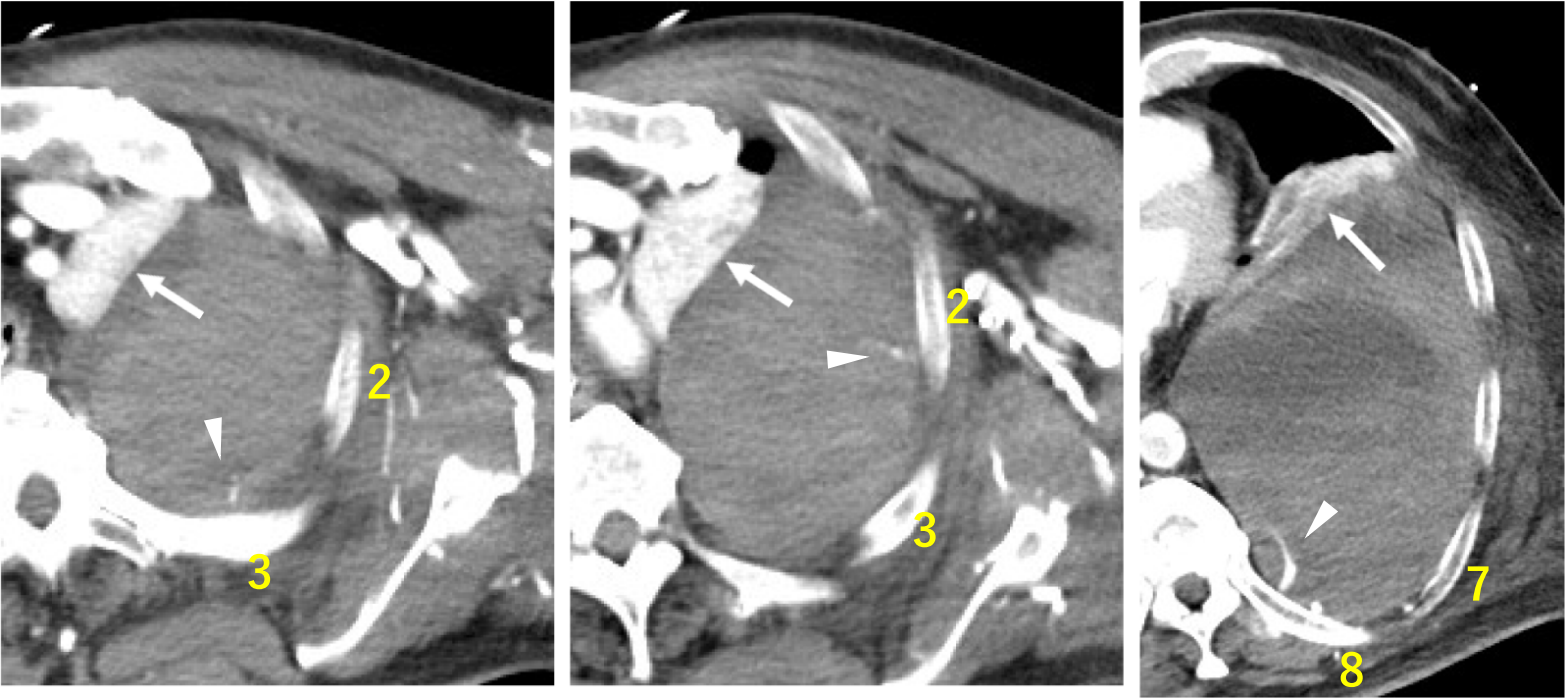
Contrast-enhanced computed tomography scan following transcatheter arterial embolization, revealing a re-enlarged extrapleural hematoma, multiple-vessel bleeding (white arrowheads), and atelectasis of the left lung (white arrows). Yellow numbers indicate the rib numbers. White arrows indicate the left lung. White arrowheads indicate contrast extravasation

However, 2 days later, blood pressure dropped, and anemia worsened. The patient had a prothrombin activity rate of 83% and an activated partial thromboplastin time of 48.4 seconds. Then, CT scans showed hematoma re-enlargement, multiple contrast extravasation sites, and lung atelectasis (Fig. 3). An emergency thoracotomy was performed owing to persistent bleeding. The large hematoma was excised, with no active arterial hemorrhage observed. Various chest wall areas exhibited bleeding, managed through ablation and hemostasis. The procedure lasted 81 min, with 2616 mL blood loss. VA-ECMO ceased on postoperative day (POD) 1. POD2 saw IABP removal, POD3 extubation, and POD4 chest drain removal. On POD37, after extended rehabilitation, the patient was discharged. A month later, coronary artery bypass grafting addressed the remaining lesions, including left main coronary artery lesion.

## Discussion

Intercostal artery bleeding can also be precipitated by medical procedures, such as dialysis, transcatheter aortic valve implantation, ultrasound-guided liver biopsy, PCI, and IABP [6, 11, 12]. In this case, the patient initially presented with three-vessel coronary artery disease identified through CAG. Subsequently, a pacemaker and IABP were implanted to manage hemodynamic instability, followed by PCI. While bleeding complications from arterial guidewire manipulation during PCI have been reported in 0.2–0.5% of patients, these incidents typically occur during the procedure [13]. In contrast, our patient experienced shock approximately 12 hours post-PCI, suggesting a different sequence of events. A similar case reported by Shiraishi *et al*. described left hemothorax and shock occurring 8 hours following IABP placement [14]. In their study, a catheter twist in the descending aorta led to perforation, resulting in bleeding from one site originating from the aorta. This contrasts with the present case, where no procedural complications were observed, classifying the bleeding as spontaneous. In this case report, the patient received post-PCI DAPT and was managed with heparin for IABP and VA-ECMO support, both during initial bleeding and after TAE. Anticoagulation has been linked to inducing spontaneous hemothorax [15, 16]. DAPT carries a higher risk of life-threatening bleeding events compared with single antiplatelet agents, and the risk escalates with heparin use [17]. Additionally, soft tissue bleeding is more prone to occur during mechanical circulatory support [18]. These facts suggest that antithrombotic therapy significantly contributed to the bleeding in this patient. Anticoagulation-related bleeding during mechanical circulatory support often proves fatal, underscoring the necessity of discontinuing antithrombotic therapy and improving coagulation function.

Previous case reports of intercostal artery bleeding, except those stemming from trauma, have predominantly featured bleeding from a single vessel, with instances of dual- or triple-vessel bleeding being exceedingly rare [1, 10]. In this patient, contrast-enhanced CT showed 15 extravasation sites suggesting bleeding in numerous vessels, prompting TAE to embolize the six intercostal arteries, which is the most commonly reported procedure to date. As the patient was presumed to have experienced continued bleeding, open thoracotomy hemostasis was required. It remains uncertain whether all bleeding originated solely from the intercostal artery of the present patient.

The primary treatment for intercostal artery bleeding is TAE, which has demonstrated relatively favorable outcomes [4, 6, 10]. On the contrary, emergency open thoracotomy has shown less favorable results [19, 20]. Emergency open thoracotomy presents challenges in identifying the precise source of bleeding during the acute phase, often necessitating subsequent reoperation. Therefore, TAE is generally favored over emergency open thoracotomy. Tanaka *et al*. proposed the surgical removal of a hematoma after stabilization with TAE [21]. Without uncontrolled or massive bleeding, conservative management may also be a viable option [22]. In the present patient, the bleeding occurred from multiple vessels, posing challenges in its management. In such cases, it may be necessary to use a combination of several treatment approaches.

## Conclusion

We report a case of spontaneous intercostal artery bleeding occurring simultaneously in numerous vessels during antithrombotic therapy with mechanical circulatory support, possibly representing the most extensive occurrence of such bleeding reported thus far. Because bleeding can occur from numerous vessels even without trauma, the findings of contrast-enhanced CT should be carefully interpreted to identify bleeding sites and determine the optimal treatment strategy. Surgical intervention may be considered if CT findings show extensive atelectasis with a massive hematoma or multiple-vessel bleeding possibly owing to nonarterial bleeding.

## Data Availability

All data generated or analyzed during this study are included in this published article.

## Abbreviations

CT: Computed tomography
CAG: Coronary angiography
IABP: Intra-aortic balloon pumping
PCI: Percutaneous coronary intervention
ICU: Intensive care unit
DAPT: Dual antiplatelet therapy
TAE: Transcatheter arterial embolization
VA-ECMO: Venoarterial extracorporeal membrane oxygenation
POD: Postoperative day
